# Enhancing liquid-chilled storage and cryopreservation capacities of ram spermatozoa by supplementing the diluent with different additives

**DOI:** 10.5713/ajas.19.0338

**Published:** 2019-10-21

**Authors:** Sherif A. Rateb, Marwa A. Khalifa, Ibrahim S. Abd El-Hamid, Hesham A. Shedeed

**Affiliations:** 1 Animal and Poultry Production Division, Desert Research Center, Ministry of Agriculture and Land Reclamation, Cairo 11753, Egypt

**Keywords:** Cryopreservation, Oxidative Stress, Computer-assisted Sperm Analysis (CASA), Ram Semen

## Abstract

**Objective:**

In the present study, we determined efficiency of incorporating caffeine, melatonin or omega-3 polyunsaturated fatty acid in the diluent on mitigating consequences of (a) liquid chilled- and (b) cryo-storage of ram spermatozoa.

**Methods:**

In the first experiment, ejaculates (n = 30) were collected from 5 adult rams and were pooled, diluted (1:10) with Tris-citric acid (base diluent) and were split into 4 aliquots assigned for: control (untreated), caffeine (0.1 mM), melatonin (0.3 mM) or omega-3 fatty acids (0.3 mM) (T_0_). The diluted specimens were stored at 4°C for 48 h, during which sperm physical and cytological properties were evaluated along with oxidative stress indices (T_24_, T_48_). In the second experiment, 15 ejaculates (3 per male) were pooled, diluted with glycerolized base diluent (4% glycerol, v/v) and were split corresponding to the same previous treatment groups before being processed for cryopreservation. Post-thaw physical and kinematic sperm properties were assessed by a computer-assisted sperm analysis system.

**Results:**

The results clarified superiority of both melatonin and omega-3 supplementation on maintaining (p<0.05) sperm properties, while reducing (p<0.05) lipid peroxidase reaction and enzymatic activities of alanine aminotransferase, aspartate aminotransferase, and alkaline phosphatase in preservation medium, compared to caffeine either during liquid-chilled storage or cryopreservation of spermatozoa.

**Conclusion:**

Melatonin and omega-3 are regarded efficient alternatives to caffeine when processing ram spermatozoa for application of artificial insemination or *in vitro* fertilization.

## INTRODUCTION

Extensive application of sophisticated reproductive techniques played a vital role in improving livestock industry in recent years. Artificial insemination (AI) and *in vitro* fertilization (IVF) are among the most applicable techniques utilized to achieve this goal in large domestic animals. In sheep, however, successful application of such technologies is still lacking although it is essentially required to develop breeds with superior productivity, resistant against diseases, or high capacity to cope with harsh environmental conditions [1]. Consequently, developing sperm processing procedures, to maintain sperm physical properties and fertilization potential, became a prerequisite for successful application of these techniques.

Liquid-chilled storage is considered most appropriate for sperm preservation when semen is required within a short period of time after collection for AI or IVF [2]. However, the full potential of semen utilization, mostly in commercial application schemes, relies on cryopreserved doses [3]. Nonetheless, both sperm preservation techniques have detrimental effects on physical and morphometric properties of spermatozoa and, hence, on sperm fertilizing capacity [4].

Sheep sperm cell membranes contain higher amounts of polyunsaturated fatty acids (PUFAs) than in other species [5]. Therefore, ram spermatozoa are highly susceptible to the oxidative damage that occurs during chilled storage [6] or freezing/thawing cycle of semen [7].

Primarily, the seminal fluid contains a wide spectrum of endogenous enzymatic and non-enzymatic antioxidants that act as free radical scavengers to protect spermatozoa [8]. However, the protective effects of such naturally-existed antioxidants decrease due to dilution of semen for processing [9]. Therefore, studies on inclusion of different antioxidants in sperm preservation medium became in focus of recent fertility-related practices. *In vitro* supplementation of melatonin [10], caffeine [11] and *n*−3 PUFAs [12] in the diluent have been reported to improve sperm preservation capacity in buffalos, rams and cow bulls, respectively. However, the degree of improvement varied dramatically among the studies due to species differences, as well as level and type of the supplement.

In the current study, two experiments were conducted to evaluate influence of incorporating melatonin, caffeine or omega-3 PUFAs in the diluent on ameliorating the oxidative stress occurring during liquid chilled and cryo-preservation of ram spermatozoa.

## MATERIALS AND METHODS

### Ethics statement

All procedures were conducted conforming to the ISO 9001: 2015 quality management regulations and were approved by the Animal Care and Use Committee of Desert Research Center, Egypt, complying with the guidelines and regulations of the Animal Ethics Committee Institute of the European Parliament for protection of experimental animals (2010/63/EU).

### Animals

This investigation was implemented at the Artificial Insemination Lab., Mariout Research Station (Latitude 31° 00′ N; Longitude 29° 47′ E), Desert Research Center, Egypt. Five sexually mature Barki rams aged 36 to 48 months, and an average body weight of 45.0±2.0 kg, were used during April, 2017. All rams were housed in a fenced stockyard throughout the period of the study, and were allowed a daily grazing period from 0800 to 1400 h. Thereafter, they were fed a concentrate mixture according to their protein and energy requirements [13]. Egyptian clover, *Trifolium alexandrinum*, hay was provided *ad libitum*, and fresh water was presented once daily after returning from the pasture. Before executing the experiment, all rams were clinically examined and were found free of disease or reproductive disorders.

### Liquid-chilled storage medium (base diluent)

A Tris-citric acid egg yolk base diluent was prepared for liquid-chilled storage of ram spermatozoa as previously reported [14]. The diluent was clarified from egg yolk particles by centrifugation at 2,400 g for 15 min and aspiration of the clear supernatant. The clarified base diluent was prepared 24 h prior to each collection session and was stored at 4°C until use.

### Semen collection

Semen was collected 3 times weekly at 0700 h by an artificial vagina. Collection tubes with modified plastic water jackets were used to maintain the ejaculates at 37°C during the collection sessions. Soon after collection, each raw ejaculate was transported to the laboratory, directly adjacent to the collection area, and was immediately evaluated for sperm physical and morphometric traits.

### Raw ejaculates assessment and pooling

The raw ejaculates were kept in a warm water bath adjusted at 37°C throughout the assessment. Ejaculate volume (mL) was recorded using the graded collection tubes. The pH, sperm concentration (×10^6^/mL), mass motility score (5 = highly motile, 0 = immotile), progressive motility (%), viability (%), normal sperm (%), and intact acrosome (%) were also analyzed for each raw ejaculate. Accordingly, adequate ejaculates of each collection session from the same rams were pooled. Mean values of the aforementioned criteria in raw-pooled ejaculates, throughout the period of the study, are displayed in Table 1.

### Experimental design

#### Effects on oxidative status and liquid-chilled storage capacity of spermatozoa

In this experiment, 30 ejaculates were obtained from the 5 rams, 6 ejaculates each, and ejaculates of each collection session were pooled and diluted (1:10) with the clarified base diluent. The diluted specimens were further split into 4 aliquots. The first aliquot served as control (untreated), whereas the other three aliquots were supplemented either with 0.1 mM caffeine (1,3,7-trimethylxanthine, Sigma- Aldrich, Shanghai, China, Cat. no. 1001176428) [15], 0.3 mM melatonin (N-Acetyl-5-methoxytryptamine, Sigma-Aldrich, St. Louis, MO, USA; Cat. no. M5250) [16] or 0.3 mM omega-3 PUFAs (Trimegavitals, Modern Health Lab. LLC, Berdsk, Russia) [14]. The later adjunct consisted of 52.6% eicosapentaenoic acid (1.65 mmol/L EPA, 20:5 *n*−3), 26.3% docosahexaenoic acid (0.76 mmol/L DHA, 22:6 *n*−3) and 21.1% α-linolenic acid (0.72 mmol/L ALA, 18:3 *n*−3). The omega-3 oil was emulsified in the egg yolk-containing base diluent and was vortexed for 2 min prior to the dilution process assuring that the oil was properly mixed with the medium. Immediately after dilution (T_0_) all specimens were transported to a cooling cabinet (4°C) and were stored for 48 h, during which sperm physical and morphometric properties were evaluated along with oxidative stress indices at 24 h interval (T_24_, T_48_).

### Semen assessment

Total sperm motility (%) was evaluated using a phase-contrast microscope (Leica Inc., Wetzlar, Germany) at 40× magnification, whereas viability was assessed by eosin-nigrosin differential staining technique at 1,000× magnification. Romanowski’s triple-stain method (DIFF-QUICK III, Vertex, Cairo, Egypt) was used to evaluate primary and secondary sperm abnormalities, as well as acrosomal cap integrity. Smears preparation and staining processes were performed as per the manufacturer’s instructions, and stained smears were evaluated by a phase-contrast microscope at 1,000× magnification. The functional integrity of sperm plasma membrane was determined by the hypo-osmotic swelling (HOS) test [17], where at least 200 sperm were evaluated at 40× magnification.

### Determination of oxidative stress indices and enzymatic activities

A portion of each semen group (2 mL) was aspirated and centrifuged (1,000 g for 10 min) at times parallel to those of sperm assessment (T_0_, T_24_, and T_48_). The aspirated supernatant was stored at −20°C until oxidative stress indices and enzymatic activities were analyzed. The changes in total antioxidant capacity (TAC), malondialdehyde acetate (MDA) concentration, reduction of the resazurin dye test (RRT) and alkaline phosphatase (ALP) activity were analyzed by colorimetric kits (Biodiagnostic, Cairo, Egypt). Alanine aminotransferase (ALT) and aspartate aminotransferase (AST) concentrations were analyzed colorimetrically by kits obtained from Spectrum, Egypt. All procedures were conducted according to the manufacturers’ instructions.

### Effects on cryopreservation capacity of ram spermatozoa

#### Cryopreservation medium and semen processing

After centrifugation and clarification as illustrated previously, the base diluent was supplemented with 2% glycerol at 37°C, and was split into 4 aliquots representing the same previously mentioned additives. Another fifteen ejaculates were collected from the same 5 rams, in 3 collection sessions, and, similar to that of experiment 1, ejaculates of each collection session were pooled and diluted (1:10) with the glycerolized base diluent (portion-A, T_0_). All specimens were equilibrated for 3 h at 4°C. Thereafter, a second aliquot of chilled-glycerolized diluent (portion-B, T_3_) was added to portion-A to reach a final concentration of 4% glycerol in whole medium. Afterwards, the specimens were equilibrated for another 2 h at 4°C (T_5_) before being packed in 0.5 mm French straws (200×10^6^ sperm/straw) using a mini-tübe filling and sealing machine (Model 133, Mini-tübe, Germany). The straws were placed in a mini-tübe biological freezer, and were exposed to nitrogen vapor (−80°C) for 10 min before being immersed in liquid nitrogen. The frozen straws were stored under liquid nitrogen surface (−196°C) until physical and kinematic properties of spermatozoa were analyzed by a computer-assisted sperm analysis (CASA) system.

### Computer-assisted sperm analysis

The frozen straws (5 per group) were thawed in a programmable thawing device (Mini-tübe GmbH, Tiefenbach, Germany) adjusted at 38°C for 40 seconds. Immediately after thawing, each sample was evaluated for sperm physical and kinematic criteria using a computer-assisted sperm analysis system (Mira-9000, Mira Lab, Cairo, Egypt). The system was designed to follow the world health organization strict criteria of human semen [18]. Prior to assessment, the system was calibrated for normal ram sperm morphometric properties and motility pattern. A minimum of 200 sperm, from 10 random bright fields, was evaluated at 500× magnification for total sperm motility (%), rapid (Class-A) and regular (Class-B) progressive motility (%), non-progressive motility (Class-C, %), immotile sperm (Class-D, %) and viability (%). Sperm kinematics in terms of straight line velocity (VSL, μm/s), curvilinear velocity (VCL, μm/s), average path velocity (VAP, μm/s), amplitude of lateral head displacement (ALH, μm), wobble movement coefficient (WOB, %), linearity (LIN, %), and straightness (STR, %) were also assessed.

### Statistical analyses

The data was checked by Shapiro-Wilk’s test and were found fitting the normal distribution. Mean values of pooled (raw) sperm properties were obtained by simple t-test. The changes in the same sperm criteria, as well as oxidative stress indices and enzymatic activities, in liquid-chilled specimens were analyzed by repeated measures analysis of variance (ANOVA) where the fixed effects of treatment, time (T_0_, T_24_, and T_48_) and treatment by time interaction were determined. Furthermore, one-way ANOVA (F test) was used to compare CASA-derived physical and kinematic sperm properties among control and treated groups. The statistical significance threshold was set at 5% and the differences between means were detected by Tukey’s post-hoc test. The data were analyzed using IBM-SPSS statistics program for windows [19]. The results are expressed as means±standard error of mean.

## RESULTS

### Effects on liquid-chilled storage capacity of ram spermatozoa

The results showed that the percent of progressive motility decreased (p<0.05) over time of storage in all groups. However, at T_48_, both melatonin- and omega 3- supplemented groups recorded the highest (p<0.05) values of progressive motility (%) compared to control and caffeine-supplemented specimens (Table 2). A similar trend was also observed in both percentages of live and normal spermatozoa (Table 2). On the other hand, no significant difference was observed in the percent of primary sperm abnormalities among control and treated groups over the storage period. However, the percent of secondary sperm abnormalities were higher (p<0.05) in control and caffeine-supplemented groups at T48 compared to melatonin- and omega 3-supplemented groups (Table 2). It is worth mentioning that the observed secondary abnormalities comprised presence of distal/translocating cytoplasmic droplets with subsequent tail opening and/or defective tails (bent, folded, or coiled tails). Contrarily, at T_48_ of storage, the intact acrosome percent was significantly lower (p<0.05) in control and caffeine-treated groups, compared to those supplemented with melatonin or omega 3. In the meantime, the control group recorded the lowest (p<0.05) percent of intact sperm cell membrane, as determined by HOS test, compared to all supplemented groups (Table 2).

### Effects on oxidative status during 48 h of liquid-chilled storage

The levels of TAC in preservation medium decreased significantly (p<0.05) in both control and caffeine-treated semen over time of storage, reaching the lowest (p<0.05) values at T48 compared to other supplemented specimens (Table 3). Contrariwise, MDA concentration increased (p<0.05) in the control group and reached the highest (p<0.05) level at T_48_ compared to all treated groups (Table 3). No significant difference was observed in reduction of RRT among control and treated specimens over time of storage (Table 3). On the other hand, enzymatic activities of ALT, AST, and ALP in preservation medium increased significantly (p<0.05) in the control group over the 48 h preservation period recording the highest (p<0.05) activities at T_48_ compared to all treated specimens (Table 3).

### Effects on post-thaw physical and kinematic sperm properties

**Figure 1:**
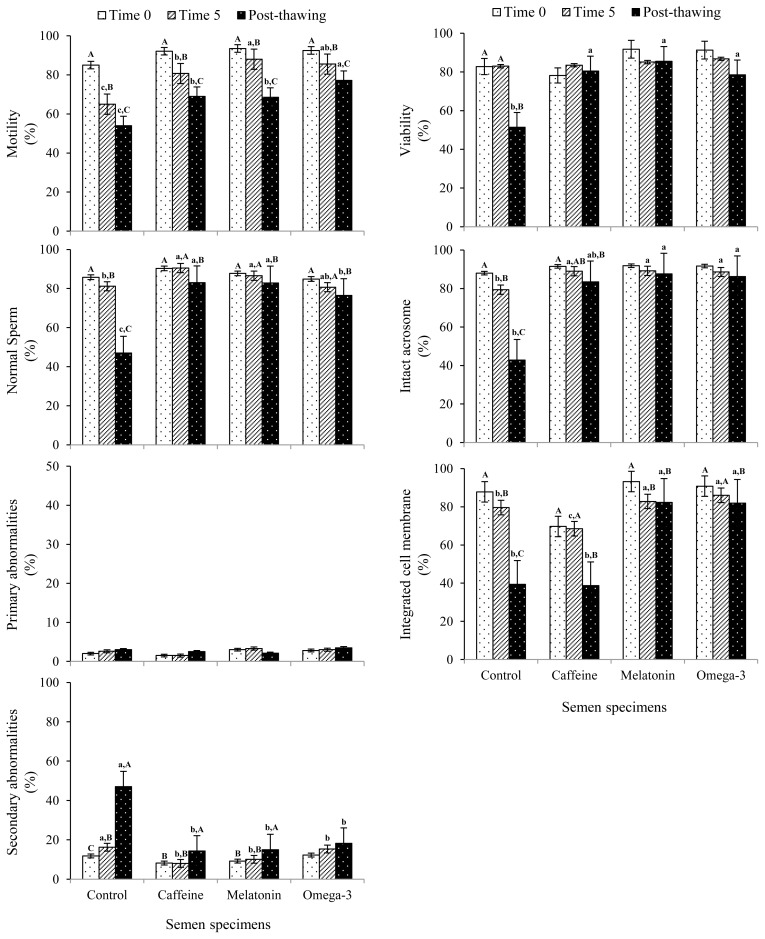
Cryosurvival characteristics of ram spermatozoa after supplementing preservation medium with different additives (mean±standard error of the mean). Control, untreated; Caffeine, medium was supplemented with 0.1 mM caffeine; Melatonin, medium was supplemented with 0.3 mM melatonin; Omega-3, medium was supplemented with 0.3 mM omega-3 polyunsaturated fatty acids (i.e. eicosapentaenoic acid [20:5 *n*−3], docosahexaenoic acid [22:6 *n*−3], and α−linolenic acid [18:3 *n*−3]). ^a–c^ Letters among groups differ significantly (p<0.05). ^A–C^ Letters within each supplement differ significantly (p<0.05).

The CASA-derived assessment revealed that the omega 3- treated specimens recorded the highest (p<0.05) post-thaw progressive motility (%), whereas the control group recorded the lowest (p<0.05) value (Figure 1). Meanwhile, the control group showed the lowest (p<0.05) percentages of sperm viability and normal spermatozoa compared to each of caffeine-, melatonin- and omega 3- treated groups (Figure 1). Even though no significant difference was observed in post-thaw primary abnormalities among control and treated groups, the percent of secondary sperm abnormalities was significantly higher (p<0.05) in the control group compared to all other treated specimens (Figure 1). Similarly, the results showed that the percent of intact acrosome was significantly lower (p<0.05) in the control group compared to all other treatments (Figure 1). Concurrently, both control and caffeine-supplemented groups exhibited the lowest (p<0.05) percentages of integrated cell membrane compared to melatonin- and omega 3- supplemented groups (Figure 1).

**Figure 2:**
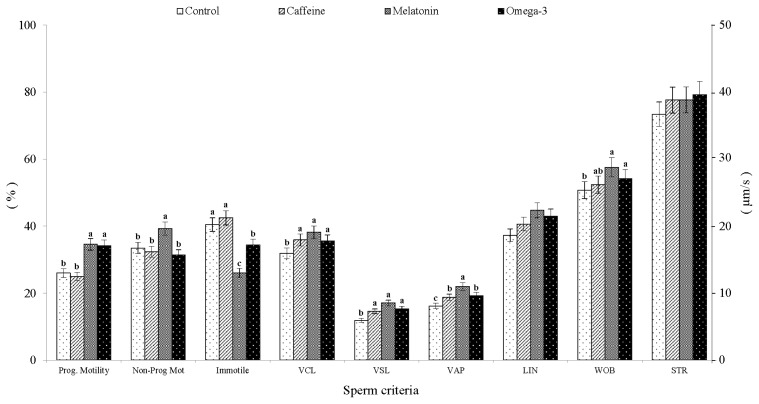
Computer-assisted sperm analysis (CASA)-derived analysis for post-thaw kinematics and velocity pattern of ram spermatozoa after supplementing cryopreservation medium with different additives (mean±standard error of the mean). Prog. Motility, progressive motility (Class-A and B, %); Non-prog mot, non-progressive motility (Class-C, %); Immotile, (Class-D, %); VCL, curvilinear velocity (μm/s); VSL, straight-line velocity (μm/s); VAP, average path velocity (μm/s); ALH, amplitude of lateral head displacement (μm); LIN, linearity (VSL/VCL, %); WOB, wobble movement coefficient (VAP/VCL, %); STR, straightness (VSL/VAP, %). Control, untreated; Caffeine, medium was supplemented with 0.1 mM caffeine; Melatonin, medium was supplemented with 0.3 mM melatonin; Omega-3, medium was supplemented with 0.3 mM omega-3 polyunsaturated fatty acids (i.e. eicosapentaenoic acid [20:5 *n*−3], docosahexaenoic acid [22:6 *n*−3], and α-linolenic acid [18:3 *n*−3]). ^a–c^ Letters among groups differ significantly (p<0.05).

The results of post-thaw sperm kinematics showed that inclusion of the three additives in cryopreservation medium affected (p<0.05) all sperm motion and velocity patterns except for motion indices of LIN (%) and STR (%) (Figure 2). In this regard, the percent of post-thaw progressive motility, particularly those of class-A and B, was higher (p<0.05) in both melatonin- and omega 3-supplemented groups compared to those of control and caffeine-supplemented specimens. Furthermore, the melatonin- supplemented group exhibited the highest (p<0.05) percent of non-progressive motility and VAP (μm/s), while recording the lowest (p<0.05) percent of immotile spermatozoa compared to all other groups (Figure 2). On the other side, values of ALH (μm) were significantly higher (p<0.05) in the omega-3 group (2.7±0.2 μm) than those observed in control (1.8±0.3 μm) and caffeine-supplemented group (1.6±0.2 μm). In addition, the results demonstrated an improvement (p<0.05) in VCL (μm/s) and VSL (μm/s) in all supplemented specimens compared to that of control (Figure 2).

## DISCUSSION

Exposing spermatozoa to cold-shock through chilled storage or cryopreservation alters their cell membrane structure and function due to spontaneous lipid peroxidase reaction (LPO) and increased MDA production. These alterations comprise redistribution of membrane bound phospholipids and proteins as well as membrane permeability and ion exchange [20], which decrease sperm viability and fertility [21] and, eventually, cause sperm death [22]. Ram spermatozoa, having high concentration of PUFAs in their cell membranes, are exceptionally vulnerable to such stress with subsequent reduced sperm motility and loss of sperm functional integrity during chilled- or cryo- preservation [2,5].

The present results clarified that caffeine supplementation drastically reduced sperm motility and increased secondary morphological abnormalities over chilled-preservation time compared to both melatonin and omega-3. This was combined with decreased TAC and elevated LPO and enzymatic activities in caffeine-treated specimens compared to the other treated groups. Furthermore, although incorporating caffeine into cryopreservation medium increased VCL, VSL, VAP, LIN, and WOB compared to melatonin- and omega 3-treated specimens, it negatively affected integrity of sperm cell membranes. This is in consonance with previous results in rams [23]. Contrariwise, improved sperm acrosome integrity has been reported after caffeine inclusion in sperm diluent of buffalos [24] and camels [25].

Caffeine (1,3,7-trimethylxanthine) is a natural stimulant belonging to the methylxanthine class, and has been utilized as a supplement in sperm capacitation medium to enhance progressive motility in IVF schemes. Caffeine inhibits cyclic nucleotide phosphodiesterase resulting in increased intracellular cyclic adenosine monophosphate. The later acts directly on sperm plasma membrane channels to increase intracellular calcium ions in sperm flagella and induces immediate sperm hyper-activation [11]. Hyper-activation is commonly observed in spermatozoa undergoing capacitation [26], which may explain the increased levels of altered acrosome/cell membranes observed in caffeine-treated specimens, particularly those subjected to liquid-chilled storage, in the current investigation. Furthermore, sperm hyper-activation implies the high energy state of spermatozoa. This was reflected in the increased sperm kinematic criteria observed in caffeine-treated specimens in the present results. Such increase in sperm kinematics could be attributed to ability of caffeine to activate glycogen phosphorylase and consequent glycogen break-down into simple sugars [27], available for consumption by spermatozoa to cope with stimulated motility.

On the other side, our results clarified the antioxidant ca pabilities of melatonin supplementation on counteracting detrimental effects of LPO reaction on chilled- and cryopreserved spermatozoa. In addition to its multiple actions on different physiological processes, melatonin and its metabolites are considered powerful antioxidants due to their ability to scavenge excessive ROS and, thus, protect spermatozoa [28]. Furthermore, melatonin has the potency to improve mitochondrial health state and functions via modulating glutathione activity thereby improving IVF outcomes [10].

The results also demonstrated the protective effects of omega-3 PUFAs on maintaining sperm physical properties and functional integrity during both liquid-chilled and cryo-storage. These results are in accordance with those reported previously in rams [14]. Omega-3 comprises a group of essential long chain PUFAs that influence biosynthetic pathways involved in regulation of animals’ various functions including reproduction. The EPA, DHA, and ALA are considered the 3 major *n*−3 PUFAs that target reproductive cells and alter reproductive function and fertility [29]. These *n*−3 PUFAs act directly on sperm membrane lipid composition to promote creation of microdomains with different flexibility, fluidity, fusogenicity, and permeability characteristics, hence, they were regarded as the major determinants of sperm mobility characteristics, cold sensitivity, viability and membrane integrity [30]. Moreover, they provide spermatozoa with energy and regulate membrane proteins thereby maintaining sperm viability during exposure to cold stress [31].

In summary, our results accentuated potency of both mela tonin and omega-3 PUFAs when incorporated into chilled- and cryo-preservation medium on ameliorating the deleterious effects of oxidative stress and, consequently, maintaining sperm physical and kinematic properties compared to caffeine supplementation. Further studies are still needed to declare the effects of inclusion of these additives in sperm preservation medium on AI and IVF outcomes.

## Figures and Tables

**Table 1:** Physical and morphometric properties of raw ram ejaculates (mean± standard error of the mean)

Parameter	
Volume (mL)	0.80±0.04
pH	7.2±0.1
Sperm concentration (×10^6^/mL)	2,335.6±22.5
Mass motility score (5-0)1)	4.28±0.06
Progressive motility (%)	92.5±0.6
Live sperm (%)	89.9±1.2
Normal sperm (%)	90.5±0.6
Intact acrosome (%)	90.1±0.9

1) Mass motility score: 5, highly motile; 0, immotile.

**Table 2:** Effect of supplementing sperm preservation medium with different additives on physical and morphometric properties of ram spermatozoa during 48 h of liquid-chilled storage at 4°C (mean±standard error of the mean)

Parameter	Preservation time (h)	Additive incorporated in medium			

		Control	Caffeine (0.1 mM)	Melatonin (0.3 mM)	Omega-3 (0.3 mM)
Progressive motility (%)	0	84.0±2.2^b^,^A^	91.7±1.7^a^,^A^	91.0±1.2^a^,^A^	93.0±0.8^a^,^A^
	24	70.0±1.0^c^,^B^	70.8±2.0^c^,^B^	79.5±1.3^b^,^B^	86.5±1.3^a^,^AB^
	48	49.0±3.1^b^,^C^	50.8±4.3^b^,^C^	75.5±3.1^a^,^B^	79.5±1.5^a^,^B^
Viability (%)	0	89.1±1.3^b^,^A^	78.2±2.1^c^,^A^	94.4±0.7^a^,^A^	93.2±0.4^a^,^A^
	24	72.8±0.6^c^,^B^	77.1±1.9^b^,^A^	86.1±0.3^a^,^B^	84.3±2.9^a^,^B^
	48	61.4±0.4^c^,^C^	69.7±2.4^b^,^B^	85.4±0.3^a^,^B^	81.6±2.7^a^,^B^
Normal sperm (%)	0	85.0±0.4^A^	90.3±1.1^A^	87.1±0.6^A^	88.2±0.6^A^
	24	71.0±0.3^c^,^B^	81.5±0.4^b^,^B^	84.7±1.1^a^,^AB^	86.1±1.9^a^,^AB^
	48	66.8±0.4^c^,^C^	72.8±0.9^b^,^C^	80.6±1.5^ab^,^B^	82.0±2.5^a^,^B^
Primary abnormalities (%)	0	2.10±0.2	2.50±0.2	2.80±0.3	2.90±0.4
	24	2.50±0.2	2.60±0.2	3.00±0.2	2.90±0.3
	48	3.70±0.4	2.50±0.2	3.50±0.6	2.50±0.3
Secondary abnormalities (%)	0	12.9±0.3^B^	7.20±1.0^C^	10.1±0.6^B^	8.90±0.6^B^
	24	26.5±0.4^a^,^A^	15.9±0.4^b^,^B^	12.3±1.2^b^,^AB^	11.0±1.8^c^,^AB^
	48	29.5±0.4^a^,^A^	24.7±0.8^a^,^A^	15.9±1.0^b^,^A^	15.5±2.3^b^,^A^
Intact acrosome (%)	0	87.1±0.9^A^	91.5±1.3^A^	91.5±0.7^A^	93.6±0.8^A^
	24	66.8±0.7^b^,^B^	87.7±2.3^a^,^A^	84.1±1.3^a^,^AB^	85.9±3.7^a^,^AB^
	48	34.0±0.9^c^,^C^	69.9±2.0^b^,^B^	79.0±1.6^ab^,^B^	81.5±6.3^a^,^B^
Intact cell membrane (%)	0	88.5±0.2^A^	79.7±1.7^A^	91.4±1.2^A^	83.3±0.3^A^
	24	63.1±1.2^b^,^B^	63.0±1.7^b^,^B^	74.9±1.1^a^,^B^	74.3±3.6^a^,^B^
	48	27.3±1.2^b^,^C^	55.8±2.2^a^,^C^	64.4±1.5^a^,^C^	59.1±5.3^a^,^C^

a–c Letters among groups in the same row differ significantly (p<0.05).

A–C Letters in the same column within each parameter differ significantly (p<0.05).

**Table 3:** Influence of incorporating different additives into ram sperm diluent on oxidative stress indices and enzymatic activities during 48 h of liquid-chilled storage at 4°C (mean±standard error of the mean)

Parameter	Preservation time (h)	Additive incorporated in medium			

		Control	Caffeine (0.1 mM)	Melatonin (0.3 mM)	Omega-3 (0.3 mM)
TAC (mM/L)	0	0.68±0.1^a^,^A^	0.53±0.1^b^	0.58±0.1^ab^	0.64±0.1^a^
	24	0.48±0.1^ab^,^B^	0.31±0.1^b^	0.52±0.1^ab^	0.64±0.1^a^
	48	0.38±0.1^b^,^B^	0.36±0.1^b^	0.64±0.2^a^	0.66±0.1^a^
MDA (nM/L)	0	17.1±1.1^B^	15.1±1.0	17.9±1.1	16.0±1.0
	24	18.7±1.8^B^	15.3±0.4	15.3±2.1	16.2±0.9
	48	26.1±2.3^a^,^A^	15.3±0.7^b^	12.9±0.5^b^	15.1±1.3^b^
RRT	0	2.50±0.6	2.70±0.4	2.40±0.9	2.00±0.8
	24	2.50±0.6	2.60±0.6	2.50±0.5	2.00±0.9
	48	2.60±0.6	2.40±0.6	2.60±0.5	2.10±0.6
ALT (U/L)	0	94.90±2.5^B^	92.7±1.3	94.6±2.7	88.1±3.5
	24	103.3±1.9^a^,^A^	91.2±2.2^ab^	87.4±1.4^ab^	78.5±1.5^b^
	48	102.1±3.6^a^,^A^	93.6±2.6^ab^	86.0±1.4^bc^	78.9±3.5^c^
AST (U/L)	0	230.6±6.9	232.1±3.2	222.3±9.7	223.8±5.4
	24	246.7±5.6^a^	238.4±3.5^b^	230.7±2.5^b^	230.3±6.1^b^
	48	261.5±6.8^a^	238.9±6.3^b^	230.2±2.6^b^	236.2±4.1^b^
ALP (IU/L)	0	243.4±3.9^B^	239.9±9.8^B^	246.1±4.8	242.1±3.5
	24	252.9±3.7^A^	251.1±6.2^A^	242.2±2.6	246.1±9.4
	48	263.9±3.6^a^,^A^	253.7±3.1^b^,^A^	245.3±4.2^bc^	240.6±9.3^c^

TAC, total antioxidant capacity; MDA, malondialdehyde acetate; RRT, reduction of resazurin dye test; ALT, alanine aminotransferase; AST, aspartate aminotransferase; ALP, alkaline phosphatase.

a–c Values in the same row with different superscript letters differ significantly (p<0.05).

A,B Values in the same column with different superscript letters differ significantly (p<0.05).
